# Oligomers and Neurodegeneration: New Evidence

**DOI:** 10.14336/AD.2023.0327

**Published:** 2023-12-01

**Authors:** Gianluigi Forloni

**Affiliations:** Neuroscience Department, Istituto di Ricerche Farmacologiche Mario Negri IRCCS, Milano, Italy

**Keywords:** Oligomeropathies, Alzheimer’s disease;, Parkinson’s disease;, β-amyloid;, α-synuclein;, neurotoxicity

## Abstract

In the last few months new results in Alzheimer’s (AD) and Parkinson’s disease (PD) have converged, attracting attention to oligomer species of misfolded proteins, β-amyloid (Aβ and α-synuclein (α-Syn), in the pathogenesis. The high affinity for Aβ protofibrils and oligomers of lecanemab, an antibody recently approved as a disease-modifying drug in AD, and the identification of Aβ-oligomers in blood samples as early biomarkers in subjects with cognitive decline, indicate the oligomers as a therapeutic target and diagnostic tool in AD. α-Syn oligomers were identified by new histopathological techniques in the hippocampus and visual cortex of PD subjects with a distribution distinct from the Lewy body pathologies but associated with cognitive impairment; these species purified from PD brain were highly neurotoxic. In a PD experimental model, we confirmed the presence of α-Syn oligomers associated with cognitive decline and sensitive to drug treatment.

In the last two decades robust evidence has increasingly pointed to the central role of small soluble aggregates, oligomers, in the pathogenesis of virtually all neurodegenerative diseases [1]. Some years ago, the term *oligomeropathies* was coined to stress the importance of this conformation in the neurodegenerative process when the insoluble aggregates are extracellular, like in Alzheimer’s disease (AD) or prion diseases, but also when the deposits are intracellular, as in taupathies or synucleinopathies [2].

In AD the presence of β amyloid (Aβ) oligomers might explain the cerebral topographic discrepancy between Aβ plaque localization and neuronal lesions as well as the scrapie-like propagation of the neuropathological lesions. The seeding mechanism responsible for protein deposition can be found in diverse cellular districts, with no substantial differences; numerous factors including pH, ionic strength, temperature, mutations in amino acid sequence and concentration can affect the amyloidogenesis [2]. Although the proteins involved in the oligomerization differ in size the chemico-physical analysis substantially confirms a common mechanism [3-6] sensitive to co-factors and metals [7], furthermore lipids have been shown to be key in the formation of oligomers and the resulting toxicity [8,9].

Oligomers are considered the intermediate structures between soluble monomers and insoluble aggregates, but this progression to mature fibrils is not inevitable: alternative oligomerization (defined as “off-pathways”) does not necessarily form larger aggregates, but the oligomer conformation remains. Moreover, oligomers can be recruited by fibrillar deposits but can also be released from them in a dynamic relationship that favors the circulation of soluble small aggregates in brain parenchyma [10]. This equilibrium among different species- monomers, oligomers, fibrils - may explain why in certain circumstances the matured fibers have been shown to exhibit some toxicity [11] (Fig.1). As underlined by numerous investigations it is reasonable to consider the oligomeric species responsible for neuronal dysfunction as a heterogeneous population rather than a single toxic conformer.

In contrast with the numerous findings accumulated at the experimental level, the presence, and the pathogenic role of oligomers in the human brain are less well understood. For instance, in an ample examination of AD pathogenesis, Frisoni et al (2022) [12] mentioned Aβ oligomers fleetingly with no specific emphasis. The interesting interpretation of clinical and neuro-pathological of AD features as stochastic event in contrast with deterministic process completely ignores the role of Aβ oligomers in AD pathogenesis [12]. The absence of direct detection by imaging techniques and the difficulties of purification of intact oligomeric conformations from brain tissue contribute to a certain level of indeterminacy. This and other observations have been used to minimize the pathogenetic role of oligomers in favor of alternative mechanism theories which would require specific investigations for proof, but in any case aliment the scientific debate [13].

**Figure 1.**
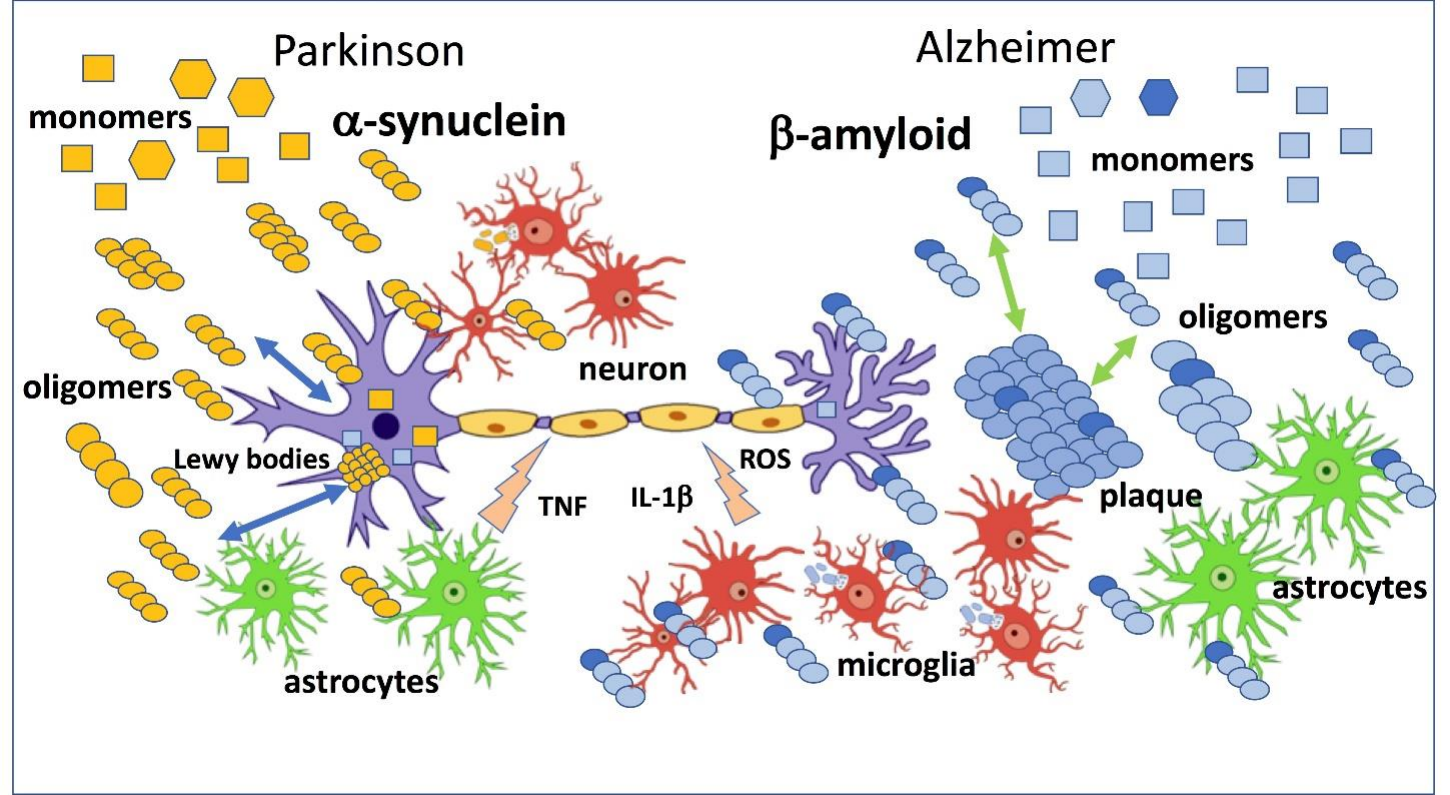
**The figure illustrates the main mechanisms associated to oligomer activities in Parkinson’s (α-synuclein, in yellow) and Alzheimer’s (β-amyloid, in light blue) diseases**. The oligomers interact directly with neurons and via glial cells activation to induce neuronal dysfunction. There is a dynamic exchange between β-amyloid oligomers and extracellular plaques in AD and between α-synuclein oligomers and Lewy bodies in PD [1, 2].

However, in the last two or three months several findings have converged to cast oligomers in a new light both in AD and Parkinson’s disease (PD). Using a primary neuronal culture model to test the toxicity of AD brain extract, Sandberg et al. (2022) [14] show that an oligomer-specific antibody (ALZ 201) depleted the toxic effect from AD brain extract. ALZ 201 recognizes only Aβ oligomers but it is equally effective in rescuing neurons exposed to AD brain extract, shown by a general Aβ antibody like 4G8 that recognize all Aβ conformers.

The success in reducing cognitive decline with lecanemab - an Aβ antibody - in AD subjects after many attempts also be related to oligomers [15]. In fact, lecanemab exhibits higher affinity for small Aβ protofibrils than other less efficient antibodies such as aducanumab or gantenerumab, also tested clinically. All three antibodies have very low affinity for the monomeric form of Aβ, lecanemab and gantenerumab have similar high affinity for the oligomeric form, but differ in their affinity to small Aβ protofibrils. Adecanumab has a good affinity only for large protofibrils and fibrils [16]. Thus, the combination of high affinity for oligomer and small protofibrils, similar in size, might help explain the clinical advantages of lecanemab and its lighter side effects than the other anti-Aβ antibodies. A clinical trial to assess the efficacy of an antibody that specifically bind soluble oligomers has been recently proposed by Siemens et al (2022) [17]. A phase 1 trial directly in AD subjects with the aim, according to authors claim, to assess target engagement and incorporate novel measure(s) to support the development of disease-modifying treatment for AD.

Recently the soluble oligomer binding assay (SOBA) has been employed to determine Aβ oligomers in AD plasma samples [18]. Using a de novo-designed α-sheet capture agent, Aβ oligomers were detected in plasma of MCI and AD patients; oligomers was found in subjects with no cognitive decline years before converting to MCI. These new observations in human samples converge to recognize an important role of soluble oligomers in AD pathogenesis and their peripheral determination as useful diagnostic tool to monitor the disease.

A chemico-physical analysis of α-synuclein (α-Syn) aggregates combined with biological and neuro-pathological investigations indicates that the oligomeric conformation of α-Syn, soluble non-fibrillar aggregates smaller than 100-200 nm, are highly toxic. Low-molecular-weight aggregates accumulated in the hippocampus and visual associated cortex in PD brains [19]. The discrepancy between the distribution of α-Syn oligomers and Lewy pathologies in PD was noted by Seklya et al (2022) [20] who confirmed the presence of α-Syn oligomers in the hippocampus of patients with cognitive impairment (Fig.1).

These important observations directly support the findings in transgenic mice carrying the A53T human mutated sequence of α-Syn [21]. This mutation is associated with early-onset PD, in transgenic mice it induced cognitive decline and an accumulation of α-Syn oligomers in the hippocampus and cortex. This evidence, apparently incongruent with PD murine models where the cognitive decline is secondary to motor impairment, is consistent with the data in humans, and substantially supports the pathogenic role of α-Syn oligomers in PD. Doxycycline abolished cognitive and daily life activity deficiencies in α-Syn A53T transgenic mice, and the effects were associated with the inhibition of α-Syn oligomerization but not with a reduction of α-Syn insoluble aggregates [21]. The findings described here confirm the prominent role of oligomers as therapeutic target in neuro-degenerative disorders.

## References

[1] Forloni G, Balducci C (2018). Alzheimer's Disease, Oligomers, and Inflammation. J Alzheimer’s Dis, 62: 1261-1276. 29562537 10.3233/JAD-170819 PMC5869993

[2] Forloni G, La Vitola P, Balducci C (2022). *Oligomeropathies*, inflammation and prion protein binding. Front Neurosci, 16: 822420. 36081661 10.3389/fnins.2022.822420 PMC9445368

[3] Jarrett JT, Lansbury PT Jr (1993) Seeding "one-dimensional crystallization" of amyloid: a pathogenic mechanism in Alzheimer's disease and scrapie? Cell, 73: 1055-1058 8513491 10.1016/0092-8674(93)90635-4

[4] Ramamoorthy A, Lim MH (2013). Structural characterization and inhibition of toxic amyloid-β oligomeric intermediates. Biophys J, 105: 287-288 23870249 10.1016/j.bpj.2013.05.004 PMC3714932

[5] Cawood EE, Karamanos TK, Wilson AJ, Radford SE (2021). Visualizing and trapping transient oligomers in amyloid assembly pathways. Biophys Chem, 268: 106505. 33220582 10.1016/j.bpc.2020.106505 PMC8188297

[6] Nguyen PH, Ramamoorthy A, Sahoo BR, Zheng J, Faller P, Straub JE, et al (2021). Amyloid Oligomers: A Joint Experimental/Computational Perspective on Alzheimer's Disease, Parkinson's Disease, Type II Diabetes, and Amyotrophic Lateral Sclerosis. Chem Rev, 121: 2545-2647 33543942 10.1021/acs.chemrev.0c01122 PMC8836097

[7] Savelieff MG, Liu Y, Senthamarai RR, Korshavn KJ, Lee HJ, Ramamoorthy A, Lim MHA (2014) small molecule that displays marked reactivity toward copper- versus zinc-amyloid-β implicated in Alzheimer's disease. Chem Commun (Camb) 50: 5301-5303. 24326305 10.1039/c3cc48473d PMC3999264

[8] Sciacca MF, Kotler SA, Brender JR, Chen J, Lee DK, Ramamoorthy A (2012). Two-step mechanism of membrane disruption by Aβ through membrane fragmentation and pore formation. Biophys J, 103: 702-710. 22947931 10.1016/j.bpj.2012.06.045 PMC3443794

[9] Pham T, Cheng KH (2020). Exploring the binding kinetics and behaviors of self-aggregated beta-amyloid oligomers to phase-separated lipid rafts with or without ganglioside-clusters. Biophys Chem, 290: 106874 10.1016/j.bpc.2022.106874 36067650

[10] Cascella R, Chen SW, Bigi A, Camino JD, Xu CK Dobson CM, et al. (2021). The release of toxic oligomers from alpha-synuclein fibrils induces dysfunction in neuronal cells. Nat Commun, 12: 1814 33753734 10.1038/s41467-021-21937-3 PMC7985515

[11] Ivanova MI, Lin Y, Lee YH, Zheng J, Ramamoorthy A (2021). Biophysical processes underlying cross-seeding in amyloid aggregation and implications in amyloid pathology. Biophys Chem, 269: 106507 33254009 10.1016/j.bpc.2020.106507 PMC10317075

[12] Frisoni GB, Altomare D, Thal DR, Ribaldi F, van der Kant R, Ossenkoppele R, et al. (2022). The probabilistic model of Alzheimer disease: the amyloid hypothesis revised. Nat Rev Neurosci, 23: 53-66 34815562 10.1038/s41583-021-00533-w PMC8840505

[13] Imbimbo BP, Ippati S, Imbimbo C, Balducci C (2022). Should we lower or raise levels of amyloid-β in the brains of Alzheimer patients? Pharmacol Res, 183: 106390. 35940397 10.1016/j.phrs.2022.106390

[14] Sandberg A, Berenjeno-Correa E, Rodriguez RC, Axenhus M, Weiss SS, Batenburg K, et al (2022). Aβ42 oligomer-specific antibody ALZ-201 reduces the neurotoxicity of Alzheimer's disease brain extracts. Alzheimers Res Ther, 14: 196. 36578089 10.1186/s13195-022-01141-1 PMC9798723

[15] van Dyck CH, Swanson CJ, Aisen P, Bateman RJ, Chen C, Gee M, et al. (2023). Lecanemab in Early Alzheimer's Disease. N Engl J Med, 388: 9-21 36449413 10.1056/NEJMoa2212948

[16] Söderberg L, Johannesson M, Nygren P, Laudon H, Eriksson F, Osswald G, et al. (2023). Lecanemab, Aducanumab, and Gantenerumab - Binding Profiles to Different Forms of Amyloid-Beta Might Explain Efficacy and Side Effects in Clinical Trials for Alzheimer's Disease. Neurotherapeutics, in press. 10.1007/s13311-022-01308-6 PMC10119362 36253511

[17] Siemers E, Hitchcock J, Sundell K, Dean R, Jerecic J, Cline E, et al. (2023). ACU193, a Monoclonal Antibody that Selectively Binds Soluble Aβ Oligomers: Development Rationale, Phase 1 Trial Design, and Clinical Development Plan. J Prev Alzheimers Dis, 10: 19-24 36641606 10.14283/jpad.2022.93

[18] Shea D, Colasurdo E, Smith A, Paschall C, Jayadev S, Keene CD, et al. (2022). SOBA: Development and testing of a soluble oligomer binding assay for detection of amyloidogenic toxic oligomers. Proc Natl Acad Sci U S A, 119: e221315711 10.1073/pnas.2213157119 PMC9897489 36490316

[19] Emin D, Zhang YP, Lobanova E, Miller A, Li X, Xia Z, et al. (2022). Small soluble α-synuclein aggregates are the toxic species in Parkinson's disease. Nat Commun, 13: 5512 36127374 10.1038/s41467-022-33252-6 PMC9489799

[20] Sekiya H, Tsuji A, Hashimoto Y, Takata M, Koga S, Nishida K, et al. (2022). Discrepancy between distribution of alpha-synuclein oligomers and Lewy-related pathology in Parkinson's disease. Acta Neuropathol Commun, 10: 13 36068646 10.1186/s40478-022-01440-6 PMC9450240

[21] La Vitola P, Artioli L, Cerovic M, Poletto C, Dacomo L, Leva S, et al. (2023). Repositioning Doxycycline for treating Parkinson’s Disease: evidence from a pre-clinical mouse model. Parkinsonism Relat Dis, 106: 105229 10.1016/j.parkreldis.2022.105229 36462409

